# Adaptive coarse-grained Monte Carlo simulation of reaction and diffusion dynamics in heterogeneous plasma membranes

**DOI:** 10.1186/1471-2105-11-218

**Published:** 2010-04-29

**Authors:** Stuart Collins, Michail Stamatakis, Dionisios G Vlachos

**Affiliations:** 1 Department of Chemical Engineering, University of Delaware, Newark, DE 19716, USA

## Abstract

**Background:**

An adaptive coarse-grained (kinetic) Monte Carlo (ACGMC) simulation framework is applied to reaction and diffusion dynamics in inhomogeneous domains. The presented model is relevant to the diffusion and dimerization dynamics of epidermal growth factor receptor (EGFR) in the presence of plasma membrane heterogeneity and specifically receptor clustering. We perform simulations representing EGFR cluster dissipation in heterogeneous plasma membranes consisting of higher density clusters of receptors surrounded by low population areas using the ACGMC method. We further investigate the effect of key parameters on the cluster lifetime.

**Results:**

Coarse-graining of dimerization, rather than of diffusion, may lead to computational error. It is shown that the ACGMC method is an effective technique to minimize error in diffusion-reaction processes and is superior to the microscopic kinetic Monte Carlo simulation in terms of computational cost while retaining accuracy. The low computational cost enables sensitivity analysis calculations. Sensitivity analysis indicates that it may be possible to retain clusters of receptors over the time scale of minutes under suitable conditions and the cluster lifetime may depend on both receptor density and cluster size.

**Conclusions:**

The ACGMC method is an ideal platform to resolve large length and time scales in heterogeneous biological systems well beyond the plasma membrane and the EGFR system studied here. Our results demonstrate that cluster size must be considered in conjunction with receptor density, as they synergistically affect EGFR cluster lifetime. Further, the cluster lifetime being of the order of several seconds suggests that any mechanisms responsible for EGFR aggregation must operate on shorter timescales (at most a fraction of a second), to overcome dissipation and produce stable clusters observed experimentally.

## Background

The Epidermal Growth Factor (EGF) receptor (EGFR) is a well-studied member of the ErbB family of receptor tyrosine kinases (RTKs), which are involved in cell fate decisions and are implicated in numerous human cancers [1]. Early studies indicated that there is a close relation between EGFR dimerization and tyrosine kinase activation [2,3]. EGF activates its receptor by altering the receptor's conformation and removing steric hindrances that prevent dimerization [4]. Upon activation, EGFR forms high-density membrane clusters presumably to amplify intra-cellular signaling and stimulate endocytosis [5], which facilitates signal transduction to the nucleus [6,7].

The mechanisms that contribute to the localization of EGFR [6,7] are complex and remain largely elusive. Single particle tracking microscopy data have shown that the membrane skeleton creates corral structures [8-11], which are responsible for the observed inhomogeneous diffusion of EGFR [12]. Thus, the receptor appears to perform Brownian motion inside a corral but also hop occasionally from that corral to a neighboring one. The nature of the interactions that create such diffusional barriers between corrals is still not fully understood. Furthermore, membrane rafts (a class of structures that include caveolae [9,10]) as well as clathrin pits [13] have been shown to localize receptor in the onset of endocytocis [8]. The nature of the forces that hold the receptors together is still subject to research.

In view of these complexities, theoretical approaches can aid in understanding the mechanisms involved in these processes and hold great potential to assist with the design of cancer related pharmaceuticals [14]. To this end, previous modeling studies on receptor clustering and inhomogeneous diffusion have shed light on the rich dynamical behavior observed in such systems. Guo and Levine [15,16] have developed a phenomenological thermodynamic model for receptor clustering, in which the latter manifests as a first-order transition, attributed to energetic interactions between receptors. Shi [17] has presented a statistical mechanical model that couples receptor clustering with signaling. In that model the interaction energy depends on the conformational state of the receptor and the presence of bound ligand. For the membrane dynamics of EGFR specifically, Mayawala et al. [18] have performed kinetic Monte Carlo (KMC) simulations for an EGFR model incorporating diffusion, ligand binding and dimerization. It was shown that the predominant dimerization pathway depends on receptor density as well as ligand concentration.

The models just discussed do not take into account spatial inhomogeneities in the cell membrane. Yet, the presence of the membrane skeleton partitions the membrane area into compartments, between which the receptor particles hop. Leitner et al. [19] have developed a stochastic corral model that captures such inhomogeneous diffusion phenomena. In that model, the segments of the cytoskeleton dissociate and re-associate acting as gates, thereby regulating protein mobility. Furthermore, Niehaus et al. [20] have analyzed the stochastic dynamics of receptor diffusion in corralled membranes theoretically and computationally, and have derived an effective macroscopic diffusion coefficient that lumps microscopic diffusion and inter-corral hops.

In the present work, we simulate membrane inhomogeneities that pertain to membrane rafts or clathrin pits. We present a simple biological model that includes receptor diffusion, reversible dimer formation, and the dynamics of cluster dissipation. Membrane diffusion barriers are assumed to separate high- from low-receptor-density areas. The barrier is assumed to be higher for dimers than monomers, thereby allowing monomers to diffuse faster out of the high-density-area. The justification for this choice is that the cell would need to keep the activated dimeric repressor into the pit to stimulate endocytocis, but the monomers could be left to diffuse out of the high-density-area. Such a mechanism is not known yet, and it is difficult to experimentally isolate the effect of dimerization, since inhibitors of dimerization also affect kinase activity [21]. On the other hand, several recent studies have shown that receptor recruitment is inefficient and internalization proceeds with slow rates for EGFR mutants that lack kinase activity [22,23] or for wild-type receptors inactivated by small molecule inhibitors [24]. It has also been explicitly argued that dimerization of EGFR is sufficient in triggering endocytocis [25]. Our model captures inefficient receptor recruitment for EGFR monomers by assuming different diffusion barriers for the monomeric and dimeric forms of the repressor. The effect of diffusion is discussed in the results and the Additional file 1.

We use the KMC method due to its ability to resolve microscopic and mesoscopic spatial (e.g., membrane rafts [26], clathrin pits [27], or corrals [12]) and temporal heterogeneity and correlations arising from reaction nonlinearities, track individual molecules, and account for a small number of molecules. Specifically, individual receptor locations and different domains of the membrane are discretely represented, and the spatial heterogeneities in receptor density and membrane environment can easily be captured. Furthermore, tracking of receptor locations allows straightforward comparisons to single particle tracking experiments.

However, biological systems' modeling covers usually wide time and space scales rendering the KMC method CPU intensive. Hence, a multiscale approach to bridge the separation of scales, while preserving the attributes of KMC, is needed. The coarse-grained (kinetic) Monte Carlo (CGMC) method [10], which groups microscopic sites into coarse cells, is a possible multiscale framework. In the CGMC method, substantial acceleration is achieved due to the reduction of the total number of cells simulated and the longer diffusion distances executed by molecules (time acceleration). However, the method treats the receptors within a coarse cell as well mixed, an assumption that naturally leads to some loss of accuracy (its error when simulating chemical reactions is though not well understood). This necessitates the use of an adaptively refined grid [28,29] to improve accuracy of the CGMC simulation. In the specific system modeled herein, a microscopic grid should be used in high-density plasma membrane areas. In order to demonstrate the source of error in coarse graining and the computational advantage of the ACGMC method, we compare for the first time the accuracy and computational efficiency of the KMC, CGMC and ACGMC methods when reactions (e.g., reversible dimerization) are considered and perform sensitivity analysis for the lifetime of an EGFR cluster. We finally discuss the biological implications of our results which may give insight into the time scale of a regeneration or recruitment mechanism of clusters of receptors.

## Microscopic model

A stochastic lattice model for EGFR dynamics is simulated, in which a membrane domain is represented as a grid of sites that may be occupied by a species. The state of the system is represented by the occupancy function , which evaluates to 1 if species *S *occupies site *i *of type *ϕ*; otherwise, it returns 0. For our simulations, a species may be a receptor monomer (M) or a dimer (D). The empty site is represented by an asterisk. For example, if , then site *i *is unoccupied. Two types of sites are utilized to represent the high- and low-density regions of the cell membrane and the hops between them.

The processes that are considered are shown in Tables 1 and 2 and amount to receptor hopping from an occupied site to a neighboring unoccupied one, receptor dimer formation from neighboring monomers, and dimer dissociation to form two monomers. This is a minimal set of key processes that can give insight into the EGFR membrane dynamics, as dimerization can be correlated to signaling rates.

**Table 1:** Diffusion model in CGMC simulations.

Description	Mechanism	Rate Constant
Diffusion in central domain	*R*_1_+ *_1 _→ *_1_*+ R*_1_	2.50 10^5 ^s^-1^
Diffusion in outer domain	*R*_2_+ *_2 _→ *_2_*+ R*_2_	2.50 10^5 ^s^-1^
Barrier diffusion	*R*_1_+ *_2 _↔ *_1_*+ R*_2_	2.50 10^2 ^s^-1^

Notation: EGF receptor (R), lattice vacancy (*), central domain (X1), outer domain (X2)

**Table 2:** EFGR diffusion-reaction model.

Description	Mechanism	Rate Constant
**Diffusion:**		
central domain (M)	*M*_1_+ *_1 _→ *_1_*+ M*_1_	2.50 10^5 ^s^-1^
outer domain (M)	*M*_2_+ *_2 _→ *_2_*+ M*_2_	2.50 10^5 ^s^-1^
central domain (D)	*D*_1_+ *_1 _→ *_1_*+ D*_1_	1.25 10^5 ^s^-1^
outer domain (D)	*D*_2_+ *_2 _→ *_2_*+ D*_2_	1.25 10^5 ^s^-1^
Barrier (M)	*M*_1_+ *_2 _↔ *_1_*+ M*_2_	2.50 10^2 ^s^-1^
Barrier (D)	*D*_1_+ *_2 _↔ *_1_*+ D*_2_	1.25 10^-1 ^s^-1^

**Reactions:**		
Monomerization	*D *+ * → *M *+ *M*	1.70 10^-2 ^s^-1^
Dimerization	*M *+ *M *→ *D *+ *	5.67 10^2 ^s^-1^

Notation: EGF receptor monomer (M), dimer (D), lattice vacancy (*), central domain (X_1_), outer domain (X_2_). Dimers occupy a single lattice site. Monomerization and dimerization reactions do not occur over a barrier.

The stochastic rates of occurrence (propensities) of the processes just noted are given as functions of the occupancy vector . Specifically, the monomer's hop propensity from site *i1 *of type *ϕ1 *to site *i2 *of type *ϕ2 *will be:(1)

The dimer dissociation (monomerization) at site *i1 *of type *ϕ1 *will be:(2)

The dimer formation (association) between monomers existing at sites *i1 *and *i2 *(of types type *ϕ1 *and *ϕ2*) will be:(3)

Once all propensities have been calculated, the random time at which the next event will occur is calculated as:(4)

where *a*_0 _is the sum of all propensities and *u *is a uniformly distributed random number. The event m to be realized at time t_current _+ τ is randomly chosen from the list of all possible events, which consists of all microscopic processes (diffusion, dimerization, monomerization) at each site. The higher the propensity of event m, the higher the probability that this event will be realized at time t_current _+ τ. This simulation scheme is similar to the Gillespie algorithm [30,31] but for processes occurring on a lattice. A noticeable difference in our implementation (from the Gillespie direct simulation method) includes a binary-tree search and update algorithm [32,33] to handle the computational cost arising from the large number of microscopic sites of a lattice.

## Simulation Setup

The plasma membrane often consists of small areas of high-density receptors in large areas of low-density receptors. In order to simulate such a system, in all simulations (unless otherwise noted) a single rectangular domain of side length equal to 48 nm was placed in the simulation space. This side length is within the generally accepted membrane raft size of 10-200 nm [34,35]. This domain represents a potentially 'high-density' region of the membrane, which accounts for 4% of the entire simulation box. Thus, for this simulation, the 48 × 48 nm domain is enclosed in a 240 × 240 nm simulation box to which periodic boundaries were implemented. A lattice constant (the distance between lattice sites) of 6 nm was chosen following [27]. The whole simulation space consists of 40 × 40 (1600) sites, and a high-density domain of 8 × 8 (64) sites. Larger domains (e.g., 1024 × 1024, representing a membrane section size of ~38 μm^2^) were also simulated and showed comparable results (not shown).

To initialize the simulation, a high number of EGFR monomers are placed within one high-density region (or within multiple regions where applicable), the latter being surrounded by an area of low-density of receptors. Consequently, most reactions will happen at short time scales in the high-density region, whereas few will occur at the low-density region.

## Monte Carlo Methods

For the simulations of this work three frameworks were used and compared: KMC, uniform mesh CGMC (hereafter abbreviated as UCGMC), and ACGMC. Exact KMC simulations fully resolve microscopic events, and can thus be extremely computationally intensive when simulating fundamental EGFR processes (diffusion, dimerization, etc.), for long times and in treating diffusion barriers from high to low-density regions. The need for long simulations arises from the necessity of simulating long-lived structures, such as clusters of receptors. To overcome these time scale issues, one has to resort to approximate methods in order to reduce the computational cost with minimal loss of accuracy.

Uniform mesh CGMC (UCGMC) simulations have been shown to accelerate simulations of systems with a wide range of time and space scales [36]. These methods refer to coarse-grained cells consisting of several sites. Hence, one introduces stochastic processes for the number of particles in a coarse-grained cells (the coarse variable), rather than resolving events at the microscopic (single-site) scale [28,29]. However, these processes exhibit error when nonlinear chemical (e.g., bimolecular) reactions occur in areas of high concentration due to averaging within each coarse cell, which results in an incorrect description of spatial correlations [28,29,37]. Thus, we expect the UCGMC method to inaccurately simulate the processes in a high-density area.

We propose that the adaptive CGMC (ACGMC) method [30] is one platform to overcome this problem by using a fully refined lattice for the high-density region only. In choosing a level of coarse graining of the low-density region one can perform UCGMC simulations at various levels of coarse-graining and compare results (e.g., dimerization rates) to those of a KMC simulation. The level of coarse-graining can then be chosen so that the error is smaller than a tolerance. The coarse grained variable that shows the number of particles of type α existing in sites of type *ϕ *for coarse-cell *C*_*k *_is:(5)

Here *q*_*ϕ,k *_is the number of sites of type *ϕ *existing in coarse cell *C*_*k*_. If *q*_*ϕ,k*_*= *1 then the microscopic events are resolved. The coarse-grained propensities are given as in the following examples. For dimer dissociation:(6)

For dimer formation:(7)

for the case where *k *= *k*' and *ϕ*1 ≠ *ϕ*2 (see Table three in [31,38] for more details on how to evaluate coarse propensities).

Simulations (not shown) indicate that the error depends on the diffusion and reaction time scales (the so called Damkohler number, Da) as well as the density of receptors. The rate of diffusion is roughly 3 orders of magnitude faster than monomer dimerization (Da < 10^-3^), and the concentration of monomers is expected to be low (fraction of occupied sites is <0.01). Under these conditions, spatial detail in the low-density region is unimportant (the system is basically well-mixed) and any level of coarse graining is expected to return a reasonably accurate rate of reaction.

**Figure 1:**
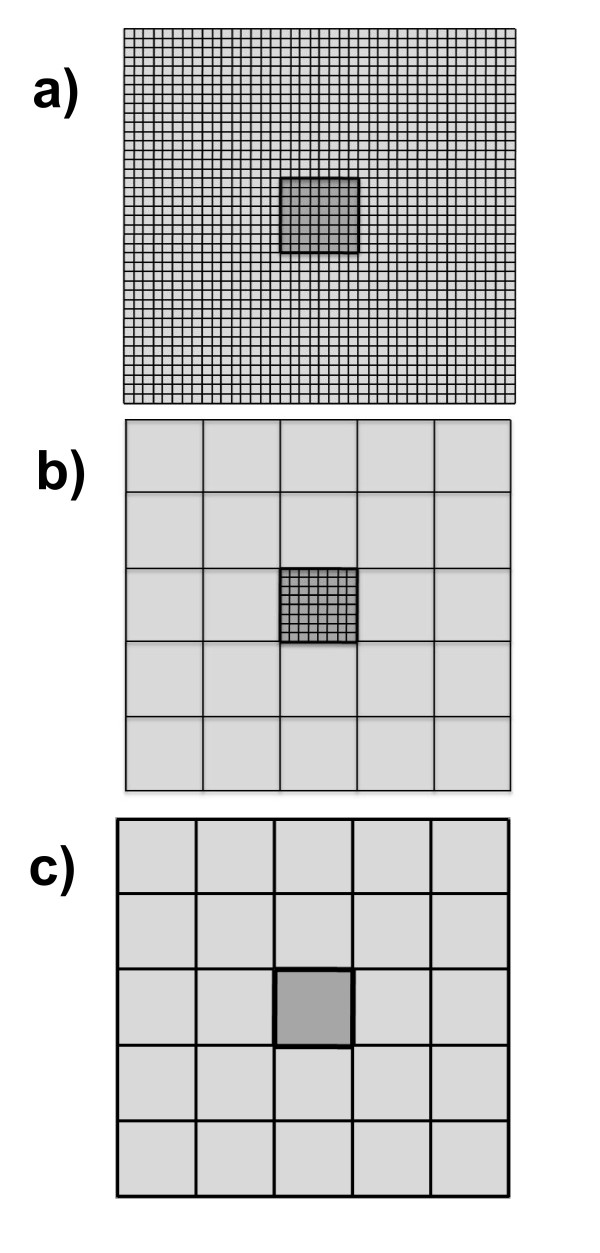
**KMC (a), ACGMC (Adaptive) (b), and UCGMC (Uniform) (c) layouts**. The central 48 nm domain starts fully covered with a local concentration of ~26000 receptors/μm^2^. A diffusional barrier separates the high-density central region from the low-density outer region.

In order to illustrate the limitations of each method, here we compare the results of the aforementioned methods, using three on-lattice simulation layouts shown in Figure 1. Panel (a) corresponds to the traditional KMC simulation, whereby all microscopic sites are resolved. Panel (c) pertains to the UCGMC simulation, whereby the entire simulation space including the central high-density region is represented as 25 coarse-grained cells of size 8 × 8 sites (48 nm × 48 nm). Finally, panel (b) portrays the grid of the ACGMC simulation, whereby the central (high-density) region is microscopically resolved, like a KMC simulation, while the rest of the lattice is uniformly coarse-grained into 8 × 8 (48 nm × 48 nm) CG cells. This multiscale approach attempts, as illustrated below, to combine the efficiency of the UCGMC method with the accuracy of the KMC method.

## Results and discussion

### Monte Carlo Simulations for EGFR Cluster Dissipation

In the CGMC method, the diffusion equations are accurate between coarse grained cells of different sizes when there is a single time scale of diffusion and there are no intermolecular forces between proteins (Fickian diffusion) [38]. However, these equations cannot be directly applied to the special case of domains separated by diffusional barriers, where the hop over a barrier is slower than the microscopic diffusion inside the domain (a two time scale diffusion process) [37]. Both the barrier and microscopic diffusion rate contribute to the effective diffusion rate between coarse cells. The effective diffusion rate between two cells separated by a single barrier, Γ_*effective*_, as a function of the barrier hopping rate and microscopic diffusion was formulated and tested in [21] and is given by(8)

**Figure 2:**
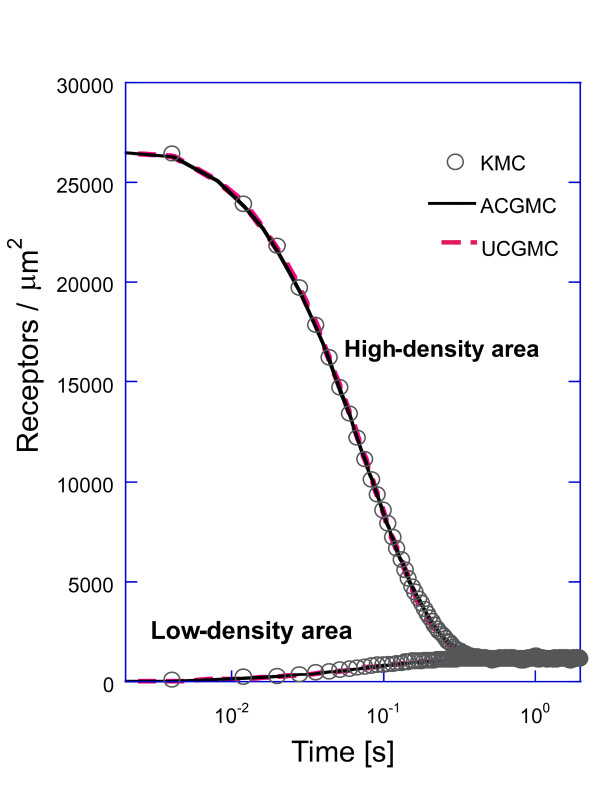
**Density of receptors vs. time obtained using three simulations for the diffusion-only model**. Both coarse-grained simulations perform accurately. For the UCGMC simulations equation (8) was used for the rate of hopping between the low- and high-density areas. Parameter values as in Table 1.

Here Γ_*micro *_and Γ_*barrier *_are the microscopic and barrier hopping rates, respectively, for a single microscopic site jump. L_CG _is the coarse cell center-to-center distance of the two relevant coarse-grained cells perpendicular to the cell boundary over which diffusion occurs. Eq. (8) was applied to inhomogeneous membrane EGFR simulations to test the accuracy of UCGMC for the case of pure diffusion (Figure 2, dashed line). Microscopic diffusion and barrier hopping occur for a single species on the lattice (Table 1). The simulation starts with concentrated receptors in the high-density region. Over time, the receptors diffuse out of this region.

The ACGMC and UCGMC methods results coincide with those of the KMC method (Figure 2), confirming that Eq. (8) correctly describes the effective diffusion rate. Additionally, this shows that the CGMC method correctly handles diffusion for spatially heterogeneous systems with high-density areas separated by low-density areas (in the absence of intermolecular forces between receptors). This allows us to attribute CGMC errors, in later high-density simulations, to reactions.

**Figure 3:**
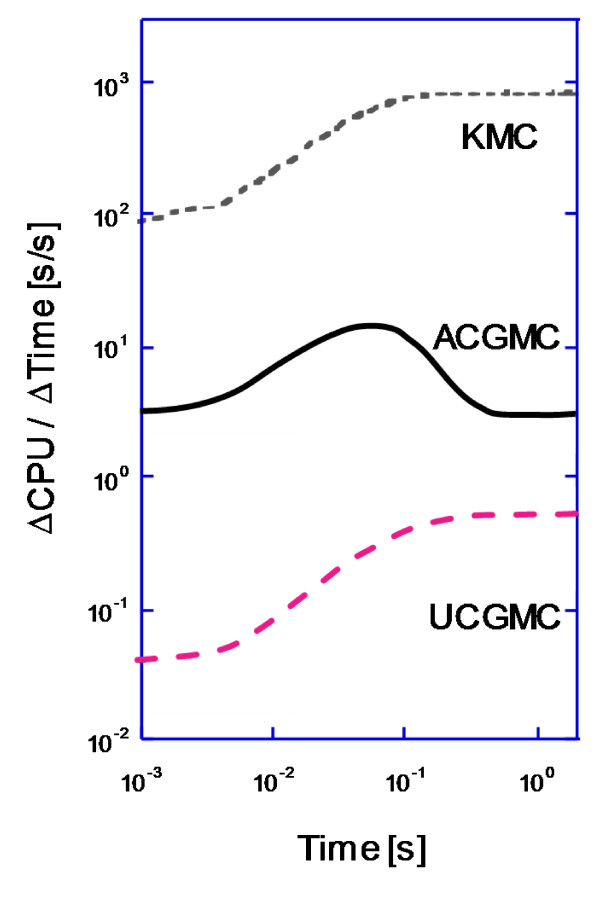
**Instantaneous ratio of CPU time to simulated time of the KMC, ACGMC, and UCGMC methods in a diffusion-only system with 48 nm × 48 nm high-density region**. Parameter values as in Table 1.

With regard to simulation cost, UCGMC simulations are cheaper than KMC by over three orders of magnitude, whereas the ACGMC method is cheaper by 0.5-2.5 orders of magnitude (Figure 3). The efficiency of the ACGMC method is especially pronounced at longer times when the majority of receptors have jumped out of the high-density region. The two coarse-grained simulations are faster because they only simulate large coarse hops in the low-density region, whereas the KMC simulation resolves all microscopic moves.

While the KMC and UCGMC simulations increase in cost until reaching a steady state, the ACGMC simulation reaches a maximum cost at a time that corresponds to the high-density region being half covered (initially it is fully covered). At this point the number of molecules and vacancies on the fine-grid, high-density region is equal, leading to the maximum frequency of expensive microscopic diffusion events. As receptors leave the central domain and the coverage fraction of occupied high-density sites falls below 0.5, the ACGMC cost drops by an order of magnitude. This is because there are fewer receptors in the expensive fine-grid central domain, and more receptors in the coarse-grained outer domain.

### Short Time EGFR Diffusion-Reaction Simulations

The previous section demonstrated that UCGMC simulations provide the same results as the KMC simulation at a much reduced cost for a diffusion only system. We expect similar CPU savings when extended to reacting systems, but the accuracy of the simulation comes into question. In the following, we investigate the performance and accuracy of the CGMC method for diffusion-reaction systems.

The reaction-diffusion model for the EGFR system is shown in Table 2. The rates are taken from Table three of [21] and represent a diffusion-controlled system. In nominal simulations, the diffusion rate of the dimer is taken to be half of that of the monomer. This choice is based on the Einstein-Stokes equation, according to which the diffusivity is inversely proportional to the radius of the diffusing particle. This may not necessarily be true in 2-dimensional diffusion on membranes, in which the dependence of the diffusivity on size is generally weaker [39]. However, as demonstrated in the Additional file 1, the results of our simulations are not sensitive to the values of these diffusivities for the parameter set chosen here.

This lack of sensitivity can be explained by identifying the rate limiting mechanism for cluster dissipation in our problem. Since the diffusivities of the receptor monomers and dimers in the inner and outer domain are much higher than the hopping rates, the rate limiting mechanism is the hopping of the monomer and the dimer across the different domains. The diffusion of monomers over the barrier separating high- and low-density regions is three orders of magnitude slower compared to the microscopic monomer diffusion inside the two regions. On the other hand, dimer diffusion over a barrier is four orders of magnitude slower than the microscopic dimer diffusion. Thus, as soon as a receptor hops out of the central domain, it quickly diffuses away, without introducing any crowding effects. Further, the values of different barrier hopping rates were chosen in accordance to intuition that once the receptors dimerize, they will be held into a clathrin pit or caveola and eventually be internalized through the corresponding endocytocis pathways. Monomeric receptors are inactive and therefore need not be internalized; instead, they are expected to diffuse easily out of the high-density domain. In the simulation, all receptors are seeded inside the high-density region in monomer form. This initial condition corresponds to the worst case scenario for the receptor cluster lifetime, namely it will give the shortest lifetimes.

**Figure 4:**
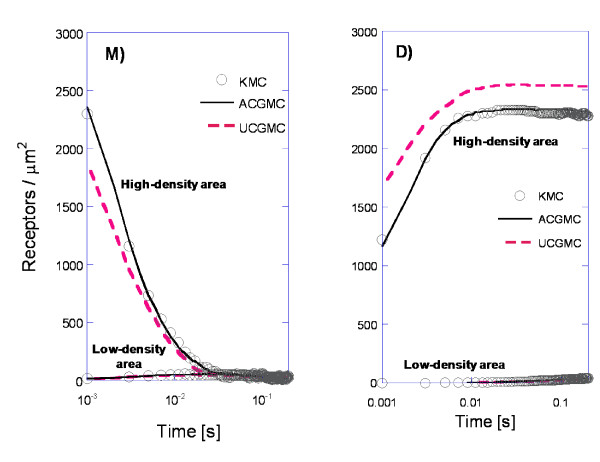
**Short-time (t = 1 - 100 ms) density of receptors in monomer (M) and dimer (D) form**. Overall density of 150 receptors/μm^2^, kinetic rates from Table 2.

Typical results for the diffusion-reaction model are shown in Figure 4. Notable is the error made by the UCGMC method due to the reactions happening inside the high-density region. At very short times (Figure 4), dimerization and monomer barrier jumps dominate. As a result, a fine grid is necessary for accurate simulations. At higher densities within the high-density region, the fine-meshes of KMC and ACGMC result in accurate but expensive simulations. At low-densities (in the outer region), the coarse meshes of ACGMC and UCGMC produces accurate results with low computational expense.

In these simulations, clusters of receptors are maintained for a while by stabilizing the dimer form inside. Initially, all receptors are in the monomer form and within a short time (<0.01 s), they either leave the high-density region or dimerize. Dimers have such a reduced rate of hopping over the barrier that the dimerized receptors are essentially locked inside the high-density area. The cluster thus formed slowly dissipates via two mechanisms: i) dimers dissociate and the resulting monomers hop through the diffusional barrier before associating again; ii) dimers hop through the diffusional barrier. Counting of the jumping events shows that the contributions of both mechanisms are of the same order of magnitude; yet, the contribution of mechanism (i) is more significant under our conditions.

### Long Time EGFR Diffusion-Reaction Simulations

The short-time simulations of Figure 4 show only the creation of receptor clusters. To calculate the lifetime of these clusters and the factors controlling cluster longevity, long simulations were performed.

**Figure 5:**
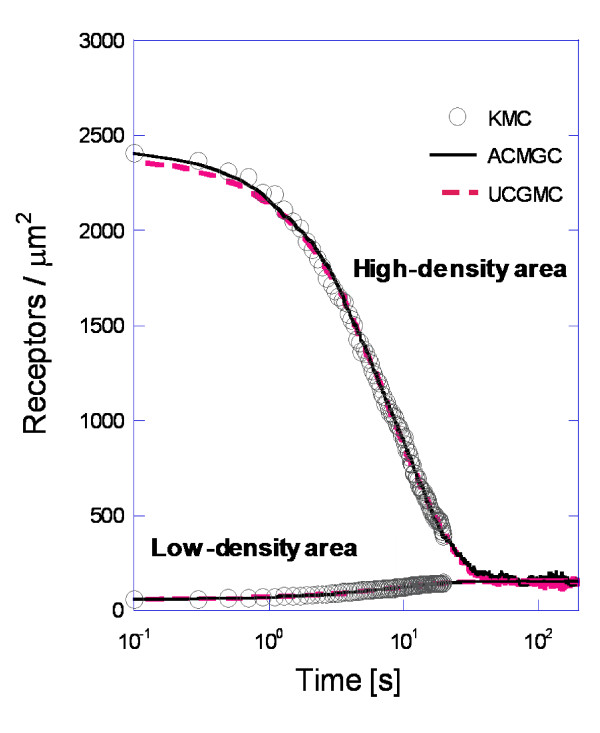
**Long-time (t = 0.1 - 200 s) trajectory of receptor density**. Overall density of 150 receptors/μm^2 ^and rates from Table 2. Dimer (two receptors per dimer) and monomers (single receptor) counts are combined.

**Figure 6:**
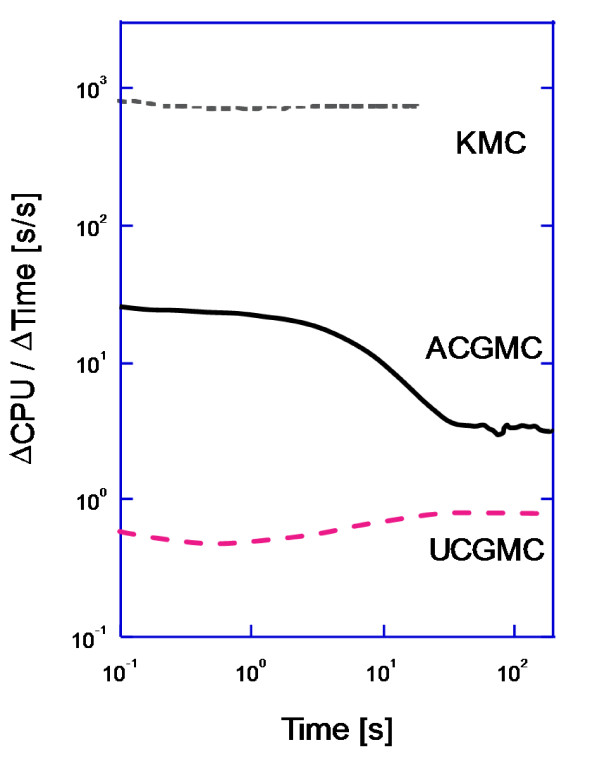
**Long-time (t = 0.1 - 200 s) CPU cost comparison of the KMC, ACGMC, and UCGMC methods in the reaction-diffusion system of Table 2 with a 48 nm × 48 nm high-density region**. This plot begins approximately at the end time of Figure 3. The ACGMC method shows an additional gain in efficiency once the corralled cluster dissipates between 10 and 100 s. KMC simulations were only run to 20 s due to computational intensity.

KMC is expensive to run at this timescale; as a result, KMC comparisons with the coarse-grained simulations were done up to only 20 s. These comparisons reveal that the ACGMC and UCGMC methods produce results that are in excellent agreement with those of the KMC simulation (Figure 5), and are able to easily reach the final steady state concentrations in reasonable CPU time (Figure 6). The accurate ACGMC simulation reduces the cost of simulation by 2-3 orders of magnitude allowing us to obtain accurate statistics and perform a sensitivity analysis discussed below.

The time courses of receptor density simulated with the ACGMC and UCGMC methods show that the monomer coverage reaches quasi-steady-state for <1 s, due to monomer diffusion between high-density and low-density regions. On the other hand, dimer hopping over the diffusional barrier and dimer dissociation is slow, and thus, the dimers are kinetically held in the high-density area and do not relax to a uniform density until ~30 s. Due to this effect, the ACGMC method becomes more efficient as the kinetically held clusters dissipate (fewer receptors in the computationally expensive high-resolution central domain) around 1 s (Figure 5). These simulations demonstrate high-density spatial receptor heterogeneity of receptors persisting on the order of seconds.

### Sensitivity Analysis

In order to investigate the dominant mechanisms controlling the properties of receptor clusters, a sensitivity analysis of the diffusion-reaction model of Table 2 was performed.

**Figure 7:**
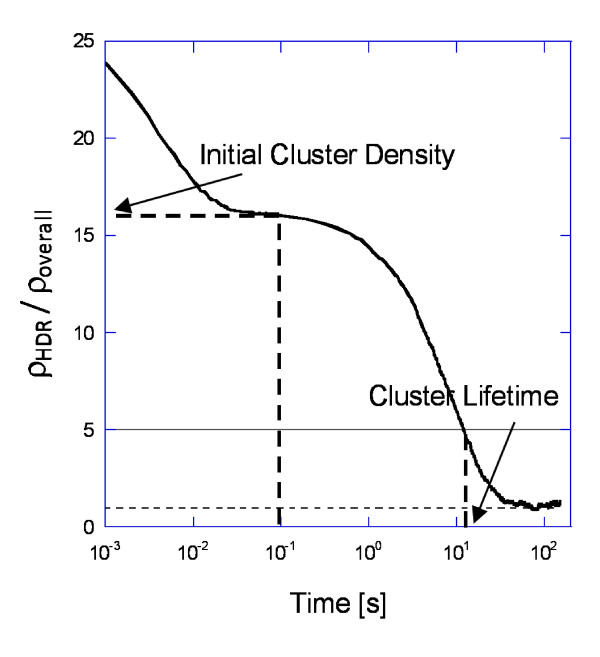
**Comparison metrics for clustering**. ρ_HDR _is the receptor density in the central (high-density) region.

To facilitate our analysis, we used two metrics, noted in Figure 7, which shows an example plot of the receptor density in the central (high-density) region (based on a weighted sum of monomers and dimers (two receptors per dimer) normalized by the overall system density (ρ_HDR_/ρ_overall_), which remains constant, vs. time. These metrics are: i) initial cluster density and ii) cluster lifetime. The initial cluster density is defined as ρ_HDR_/ρ_overall _at 0.1 s. At this time, the initial monomers have either dimerized or left the high-density area (Figure 4). This metric can also be thought of as the effectiveness of receptor trapping by the diffusional barriers when dimerization partners are readily available. The cluster lifetime is defined as the time at which the concentration of receptors within the high-density domain drops below 5 times the overall receptor density (namely ρ_HDR_/ρ_overall _= 5). The cluster lifetime illustrates how effective the diffusional barrier is at stabilizing receptors in the dimer form.

These metrics were calculated for a range of values of the following variables: size of the high-density region, overall receptor density, and dimer barrier hopping propensity. The sizes (48 nm and 24 nm) were chosen within the observed 10-200 nm range of membrane rafts on living cells [21]. The overall density of receptors was also varied, since the dramatically different density of receptors in cancerous and normal cells is suspected to play important role in the dysregulation of cell communication. Finally, dimer barrier diffusion was disabled in some simulations to reflect that dimers may cross the barrier with an extremely low probability.

**Figure 8:**
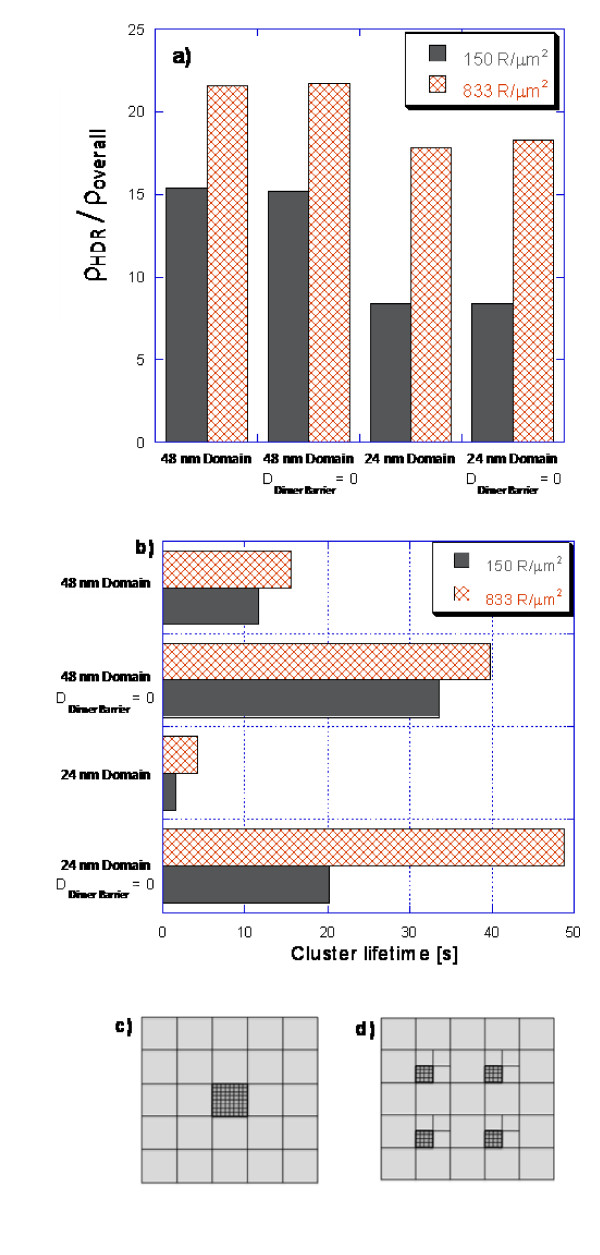
**Sensitivity of clustering to monomer-only barrier diffusion, different central domain sizes, and different overall receptor densities**. (a) Initial cluster density (ρ_HDR_/ρ_overall _at t = 0.1 s). (b) Cluster lifetime (time when ρ_HDR_/ρ_overall _= 5). (c) ACGMC layout of 48 nm domain simulations. (d) ACGMC layout of 24 nm domain simulations. All receptors initially start at random locations in the central domains.

The results of the sensitivity analysis on these metrics appear in Figure 8. The initial cluster density (Figure 8a) is most noticeably affected by the density of receptors in the simulation. For a fixed size of the high-density region, higher overall density simulations (833 receptors/μm^2^) exhibit more pronounced initial clustering relative to low-density simulations. This result can be attributed to the higher receptor density increasing the number of dimerization partners adjacent to any given monomer (higher dimerization rate). Consequently, a higher proportion of receptors remain in the dimer form as the density of receptors in the central domain increases.

Smaller domain sizes have a higher circumference to area ratio and therefore result in a higher likelihood for a monomer to border an edge (increased monomer barrier hopping rate). The diffusion time from the center of the domain to the circumference also drops. As the above logic would suggest, smaller domains have a lower initial cluster density at the same overall densities as larger domains.

Disabling dimer barrier diffusion (Figure 8a) has very little effect on the initial cluster density, reflecting that monomer barrier hopping is effectively the only path by which receptors leave the high-density region in the very early stages of the simulation. On the other hand, when dimer diffusion is taken into account, the smaller clusters have a much weaker hold on the receptors (Figure 8b). Since the former have a higher circumference to area ratio, dimers capable of jumping have more contact with the barrier and thus hop over the barriers at a faster rate, in contrast to the case of larger clusters.

Disabling dimer diffusion extends cluster lifetimes (Figure 8b) by half an order of magnitude in 48 nm domains and one order of magnitude in 24 nm domains. This disproportionate increase in small domains is attributed to a higher chance of dimerization of dissociated dimers. We assume that past the initial stage (>0.01 s of Figure 4) the probability of more than 2 monomers at a time existing in the high-density domain is negligible (since 2 monomers will most likely dimerize or jump a barrier long before another dimer breaks). Smaller domains (24 nm) will hold the two monomers much closer together than the larger domains (48 nm) giving the monomers a higher chance of association before one monomer hops over the diffusional barrier.

It appears that for large clusters, the receptor density plays a secondary role in the cluster lifetime (Figure 8b). On the contrary, for small clusters the density of receptors is a major factor for determining longevity. This suggests that the sensitivity of cluster lifetime to receptor density is correlated with cluster size. For large clusters, the size itself has a dominant effect on cluster lifetime, whereas for smaller clusters the lifetime is primarily a function of receptor density. Manipulating the cluster size together with receptor density has a greater overall effect on the dispersion rate of EGFR clusters than changing each variable individually.

**Figure 9:**
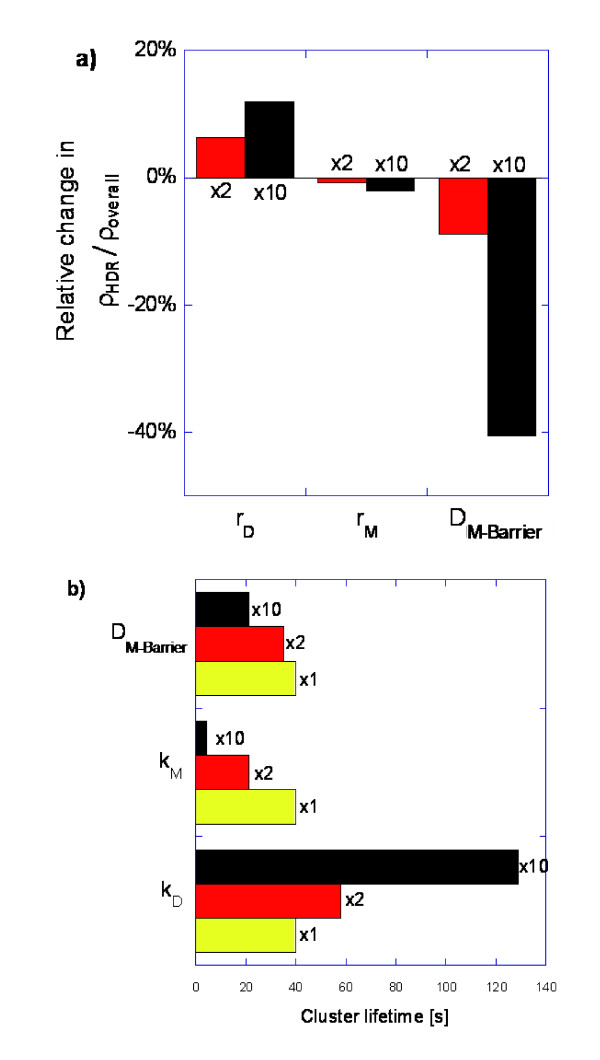
**Sensitivity of clustering to reaction and barrier diffusion rate constants**. (a) Initial cluster density (ρ_HDR_/ρ_overall _at 0.1 s). (b) Cluster lifetime (time when ρ_HDR_/ρ_overall _= 5).

In order to understand the effect of rate constants for monomerization, dimerization, and monomer barrier hopping (k_M_, k_D_, and D_M-Barrier_, respectively), we defined a new nominal case with an overall receptor density of ~833 receptors/μm^2^, disabled dimer barrier hopping, and use a side length of 48 nm for the rectangular high-density region (Figure 8c). The results of this sensitivity analysis are shown in Figure 9. Each rate was increased by a factor of 2 or 10.

It was observed that the initial cluster density is mostly affected by increasing the rate at which monomers hop over the barrier. Initially, all receptors are in monomer form, and thus, an increase in the monomer barrier jumping rate significantly decreases the initial cluster density. Similarly, higher dimerization rates result in higher initial cluster densities, since monomers lock into the dimer state faster. The rate of monomerization (dissociation of dimers) has little influence, which is to be expected, since at short times the primary events taking place are dimerization reactions and barrier hopping.

Moreover, all kinetic rate constants affect the cluster lifetime. Changes to the rate of monomerization and dimerization influence the lifetime more than changes to the monomer barrier diffusion rate. Increasing the dimerization rate by an order of magnitude increases the kinetic lifetime of the cluster by about half an order of magnitude and well into the range of minutes. Increasing the rate of monomerization by an order of magnitude shortens the cluster lifetime by approximately an order of magnitude. The increase in the rate of monomerization seems to have a relatively *greater *effect on cluster lifetime than proportional changes to the rate of dimerization.

These results can be explained as follows. More time spent in monomeric form directly correlates with faster cluster dissolution. This is because two monomers are only capable of associating to form a dimer if they both reside in the high-density region long enough for a dimerization event (a bimolecular reaction) to occur. If either monomer resulting from dimer disassociation (a monomolecular reaction) leaves the high-density region, the remaining monomer will most likely leave this region as well. This causes the overall probability of dimerization of two monomers to be a function of both the rate of monomer barrier hopping and the dimerization reaction rate constant, whereas the rate of monomerization is only a function of the monomerization reaction rate constant.

Given the uncertainty in kinetic and diffusion rate constants, it is quite possible that regions of high concentration of receptors could kinetically lock clusters over the period of minutes. Eventually, though, without a thermodynamic stabilization or regeneration mechanism, no long-term (beyond seconds to minutes) clustering will be observed with this model. Stabilization of these clusters would be necessary in order to effectively trigger endocytocis. The results of our simulations postulate that any stabilization or regeneration mechanism must operate on shorter timescales (possibly fraction of seconds or even shorter), to overcome dissipation and result in stable clusters seen experimentally.

## Conclusions

Adaptive Coarse-Grained kinetic Monte Carlo (ACGMC), a multiscale spatial stochastic simulation, was applied to the EGFR diffusion-reaction system. The ACGMC method properly captures the detailed spatial reactions in high receptor density regions of the membrane while efficiently and accurately simulating the low-density areas of the membrane with a low resolution grid. A sensitivity analysis of the density and longevity of these clusters was carried out.

Given the uncertainty in the kinetic parameters of receptor chemistry, kinetic stabilization of EGFR clusters on the order of minutes is quite plausible. EGFR clusters would only need to exist on the order of seconds or less to successfully pass on signals, or for the formation of endosomes. Our work demonstrates that diffusion barriers provide a plausible mechanism for locking clusters for short times. Any stabilization or regeneration mechanism must operate on shorter timescales (possibly fractions of seconds or even shorter), to overcome cluster dissipation. In view of our results, methods to break, strengthen, reorganize, or otherwise manipulate membrane barriers that localize trans-membrane receptors will be valuable to develop.

At relatively high receptor densities, smaller domains exhibit stronger clustering than large ones, whereas at low receptor densities clustering is weaker in smaller domains. Our results demonstrate that cluster size must be considered in conjunction with receptor density, as they synergistically affect EGFR clustering. It would thus be inappropriate to compare the behavior of cells of similar receptor densities but with different high-density region sizes or dispersions.

The ACGMC method is promising for a wide variety of multiscale and spatially heterogeneous problems. For example, it could also be conceivably expanded to three dimensions and applied to a whole new class of systems. Using the EGFR system as an example, the lifetime of an endosome, including receptor clustering, budding, and cytoplasmic transport may be simulated using 'thin' coarse cells for the membrane and coarse 3D cells representing the cytoplasm. ACGMC would allow detailed spatial resolution for high-reaction locations (local areas of the membrane), with coarse and computationally cheap cells for diffusion-heavy processes (cytoplasmic transport).

## Authors' contributions

SC developed the algorithm, set up and performed the simulations and drafted the manuscript. MS revised the manuscript, wrote the majority of the methods sections, and assessed the sensitivity of the results. DGV conceived the study, and participated in its design and coordination and helped refine the manuscript. All authors read and approved the final manuscript.

## Supplementary Material

Additional file 1 **Supplementary material**. Simulation results showing that the conclusions of this study are not sensitive to the diffusivities of the monomer and the dimer in the central and outer domains of the interval. Click here for file
