# Supplementary material for: Adaptive coarse-grained Monte Carlo simulation of reaction and diffusion dynamics in heterogeneous plasma membranes
Source: BMC Bioinformatics. 2010 Apr 29;11:218. doi: 10.1186/1471-2105-11-218 (PMC2868014; doi:10.1186/1471-2105-11-218)
Supplement: Additional file 1 — Supplementary material. Simulation results showing that the conclusions of this study are not sensitive to the diffusivities of the monomer and the dimer in the central and outer domains of the interval. [file 1471-2105-11-218-S1.DOC]

# adaptive coarse-grained monte carlo Simulation of reaction and diffusion dynamics in Heterogeneous plasma membranes

Stuart Collins, Michail Stamatakis, and Dionisios G. Vlachos

Department of Chemical Engineering

University of Delaware, Newark, DE 19716, USA

**SUPPLEMENTARY MATERIAL**

The following plots show that the simulation results are not sensitive to the diffusivities of the monomer and the dimer in the central and outer domains of the interval. For the black curves the nominal parameter set was used in which the diffusivity of the dimer is half that of the monomer. For the red curves the diffusivities of monomer and dimer are the same. The curves are hardly distinguishable. The overall density of receptors is 833 receptors/μm^2^ and the geometry is that of Figure 1b of main text.
